# Free Volume and Water Sorption by Cellulose Esters

**DOI:** 10.3390/polym13162644

**Published:** 2021-08-09

**Authors:** Anatoly E. Chalykh, Ivan I. Bardyshev, Tatiana F. Petrova

**Affiliations:** Frumkin Institute of Physical Chemistry and Electrochemistry Russian Academy of Sciences (IPCE RAS), 31, bld.4 Leninsky Prospect, 119071 Moscow, Russia; bardyshev1945@mail.ru

**Keywords:** diffusion, sorption of water vapours, double sorption model, cellulose acetates, cellulose ester structure, free volume, positron annihilation, polymer plasticisation, hydrate numbers of functional groups

## Abstract

The results ofthe sorption properties of cellulose acetate (CA) with different degrees of substitution (SD) are summarised. It has been shown that the sorption capacity of CA in water vapour decreases naturally with increasing content of acetate groups in monomeric units of cellulose ethers. The experimental isotherms are analysed according to the double sorption model. Hydrate numbers of hydroxyl and acetate groups were determined. The paired parameters of the Flory–Huggins interaction were calculated. It is shown that the decrease of the Langmuir component is due to the replacement of hydroxyl groups by ester groups, whose local sorption capacity by water vapour is lower than the sorption capacity of OH groups. In the area of high humidity, there is an increase in vacancy sizes due to plasticisation of the sorbents.

## 1. Introduction

Among currently developed models [1,2,3,4,5,6] for the description of sorption and solubility of water in polymeric sorbents, the concept of van Krevelen group contributions [7,8] based on notionsonhydrate numbers of functional groups of monomer links of macromolecular chains is widely used. The efficiency of this approach was demonstrated by describing the sorption properties of polyvinyl alcohol (PVA) [9], aliphatic and aromatic polyamides [10,11], elastomers [11], polyacrylates [12,13], polyurethanes [14], chitin and chitosan [15,16], naturally and artificially aged polyolefins [17], etc.

In [9,10,18], it was proposed to use this approach for the determination of structural and morphological features of sorbent structure: crystallinity degree, availability of functional groups of monomer links, content of hydrophilic polymers in mixed compositions and grafted systems [19], and estimation of osmotic pressure in swollen polymers [1]. In [20], an attempt was made to extend this concept to other polar sorbates, in particular, sulfur dioxide, carbon dioxide, and saturated hydrocarbons.

At the same time, for a number of polymeric sorbents—statistical and block copolymers, heteroarylenes, and aromatic polyamides, there is a deviation from the values of hydrate numbers standardised by van Krevelen [7,8]. In [10,11], a partial screening of the amide group by an alkyl radical and, as a consequence, a decrease in the hydrate number, especially in the area of high humidity, was shown for aliphatic polyamides. A similar effect is described in [18] on the example of aramid fibres.

Meanwhile, a sufficiently large amount of information on sorption isotherms for a variety of polymeric membranes, film materials, coatings, and fibres have been accumulated in recent years [1]. All this requires the specification of numerical values of hydrate numbers, development of approaches for application of this concept to glassy and crystalline states of sorbents, estimation of possibility of its use for description of water vapour adsorption on surfaces of polymers, carbon fibres, porous membranes, and grafted and modified materials.

This paper presents data on water sorption by cellulose acetates of different substitution degrees. The main attention is paid to the study of the influence of the hydroxyl group environment within a monomeric link on its availability and local sorption capacity. For this purpose, we performed a special study to determine the free volume of the sorbent in different regions of sorption isotherms.

## 2. Experimental

### 2.1. Objects and Methods

The industrial samples of cellulose acetate (CA) and cellulose triacetate (CTA) produced by OAO “Acetate” (Engels, Russian Federation) of different degree of esterification were used as objects of research (Table 1). Films with thickness from 40 to 100 µm were produced by spreading the 5% polymer solution in dimethylformamide (DMF, Rushim, Moscow, Russian Federation), which is a mixture of dichloromethane (DCM, Rushim, Moscow, Russian Federation) and ethanol (Rushim, Moscow, Russian Federation) onto a glass substrate and further vacuum drying at a stepwise increase of temperature from 333 to 390 K during several days until constant weight. According to pyrolytic mass spectrometry data, the residual solvent content was from 0.5 to 1.0 wt% for DCM and from 1.0 to 1.5 wt% for DMF. The use of cellulose ethers of different degree of acetylation as sorbents makes it possible to estimate the influence of bulk ether groups on the stability of intra- and intermolecular hydrogen bonds, to establish the ratio of hydrate and acetyl radicals, and to determine the role of free volume in the sorption capacity of cellulose ethers.

The substitution degree of acetylation of the esters (*γ*) was determined by acetic acid titration after alkaline saponification of CA according to recommendations [21]. The molecular weight of cellulose acetates was determined viscosimetrically using solutions of CA in acetone and the Mark–Kuhn–Hauvinck equation [η] = 1.6 × 10^−4^ *M*_η_^0.82^ [22]. The glass transition temperature of CA samples was determined by DSC [9]. The measurements were carried out on a NETZCN DSC 204 F1 Phoenix (Netzsch, Selb, Germany) in the temperature range from 293 to 520 K. All experiments were performed on sample weights not less than 15 mg. The results were automatically processed using Proteus Analysis, where the value of Tg was determined as the inflection point of the temperature dependence of the heat capacity curve. The heating rate was varied in the range from 10 to 20 deg/min.

### 2.2. Sorption Measurements

The sorption measurements were carried out by the traditional method [21] in the relative humidity (*p*/*p_s_*) range from 0.10 to 0.98 using a Mc Bain–Bakr balance with quartz spirals of 1mg/mm sensitivity and an optical registration system. The interval and integral measurement techniques were used [21]. Experimental results on the kinetics of water sorption in the studied polymers were obtained in isobar–isothermal modes of the processes.

The change in the sample mass in the sorption process *M_t_* was recorded using the same cathetometer by spiral elongation with an accuracy of ±0.01 mm, which provided an accuracy of sample mass measurement of ±10^−5^ g. The measurements were carried out until the sorption equilibrium was established.

Particular attention in the work was paid to the two regions of *p*/*p_s_*, which is characteristic of the low vapour activity and the inflection area on the *S*-shaped sorption isotherms. In these areas of isotherms, the step in the experiments on interval sorption varied from 0.02 to 0.05 rel.u. Thus, obtained data on the dependence of sorption capacity of samples *φ* = (*M*_∞_ − *M_o_*)/*M*_∞_ wt% on vapour activity were used for the construction of sorption (desorption) isotherms.

Statistical processing of numerous data on sorption isotherms and kinetics of sorption equilibrium establishment performed in the framework of the traditional approach [22] showed that under the chosen conditions of the experiments:

- Temperature control accuracy was ±0.05 °C;
- Weighing error was ±0.01 mg;
- Time registration error was ±5 s;
- Error in determining pressure was ±0.03 rel.u;
- Error in determining the sample thickness was ±5%.

The total error in determination of the sorption capacity was less than 5%.

To calculate the diffusion coefficients [23,24], we used the traditional Fick equations for the kinetics of water vapour sorption by the CA membrane of thickness *L*:

(1) MtM∞=γ=4π1/2×(DtL2)1/2

(2) MtM∞ =1−∑{8(2n+1)2π2exp(−D(2n+1)2π2tL2)}

where *M_t_* is the mass of the sample at time *t*; *M_t_* is the time-invariant mass of the sample at constant vapour pressure *p* and temperature *T*. *M_∞_* was assumed as the value of *M_t_*, which remained unchanged for a time twice as long as the time of equilibrium establishment; *D* is the partial coefficient of diffusion of water vapour, cm^2^/s, *γ* is the degree of filling of the sorbent with sorbate, *t* is time. Statistical processing of the experimental data on the sorption kinetics showed that the relative error in determination of the diffusion coefficients using the interval experimental method was 5–8%.

### 2.3. Positron Annihilation

To determine the free volume of CA (vacancy rate) samples and its change during filling with sorbed water molecules, the positron annihilation (PA) method was used. The PA method is based on the property of positrons to recombine with environmental electrons and emit hard gamma radiation [24,25]. It is known that the annihilation characteristics of the recombination process, the positron lifetime (PLT), and the angular correlation (AC)of paired gamma quanta are determined by the electron density and distribution of the electron pulses at the annihilation site.

In polymeric materials, a significant fraction of positrons during deceleration in a polymeric sorbent forms so-called positronium atoms (Ps), which are a bound “positron–electron”system that is chemically similar to the hydrogen atom [24]. During diffusion, *Ps* atoms localise (absorb) in regions of free vacancy volume and annihilate from the trapped state. The PLT and AC of the trapped Ps atoms differ from those of Ps annihilating in the polymer matrix. Therefore, by isolating the corresponding components from the experimental PLT and UC spectra, one can obtain quantitative information on the size and concentration of the vacancies—Ps atom traps [26].

**Figure 1:**
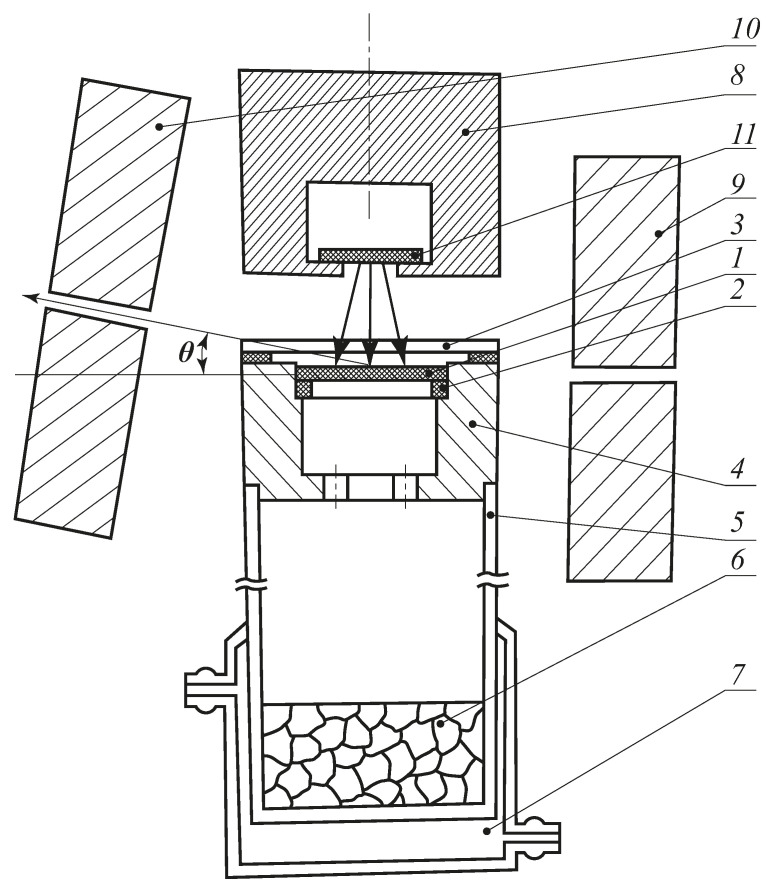
Setup for positron angular correlation curves measurement: 1—studied polymer film, 2—thin aluminum ring, 3—mica window, 4—chamber, 5—glass vessel, 6—vapour source, 7—jacket, 8—lead shielding, 9,10—fixed and movable lead collimators, 11—positron source.

The measurements were carried out on the AC setup with a parallel-slit geometry with angular resolution σ* = 1 mrad. The Na^22^ isotope with an intensity of 0.1GBq was used as a positron source. To measure AC curves in thin films, a special cell was developed, which allows the experiment to be carried out under isothermal conditions in an atmosphere of water vapour with a given humidity. The scheme of the cell is shown in Figure 1.

The studied polymeric CA film was fixed onto a thin aluminum ring (2), placed in a chamber with aluminum walls, and sealed from above with a mica window (3), through which positrons emitted by a radioactive source (11) penetrated. The thickness of the mica window of 1.8 mg/cm^2^ provided free passage of more than 90% of the positron flux through it. The chamber with the sample was hermetically connected with a glass vessel, in which an aqueous solution was placed, which provided the specified humidity in the chamber volume during the whole process of the angular correlation curves measurement (about 20 h). To perform the measurements on dried polymer samples, a CaCl_2_ desiccant was placed in the vessel. The lower part of the vessel was equipped with a jacket that allowed the cell to be thermostatted during the measurements, if necessary.

The cell was placed in the setup for angular correlation curves measurement directly under the positron source and was placed in such a way that the studied polymer film was in the field of view of the slits of the setup, so that only the annihilation gamma quanta of those positrons that were decelerated and annihilated in the studied film were registered. The lead shielding of the positron source and lead collimators excluded the registration of annihilation radiation of the source itself and positrons that passed through the studied film as well as the positrons annihilating in the mica window and in the chamber walls.

The study technique included a preliminary drying of the sample at a humidity of less than 0.1%, which was followed by stepwise saturation at different humidities to an equilibrium value. At each *p*/*p_s_* value, after sorption equilibrium was reached, the dependence of the count rate of paired annihilation quanta (with an energy of 511 keV each) on the angle of their dispersion *θ* (the gamma–quantum pair deviation angle from 180°) was determined.

To determine the free volume parameters in the polymer films, a “narrow” component related to the annihilation of para-positronium atoms (*p*–Ps) localised in micropores was isolated from the experimental AC curves. A modified Trumptu method [25] was used to isolate the “narrow” component. The “narrow” component was defined as the difference between the experimental AC curve and the “broad” component obtained by Gaussian approximation in the region of gamma ray angles of *θ* > 5 mrad. The difference curve *C_N_*(*θ*) in the region of angles of 0 < *θ* < 5mrad was represented as the ‘narrow’ component. Furthermore, its half-width FWN (full width at half-height) and intensity *I_n_* (ratio of FWN area to total curve area) were determined. Based on the FWN and *I_n_* values obtained, the effective mean radius *R_v_*, and concentration *n_v_* of micropores, Ps atom traps were calculated in the spherical shape approximation [27]:

(3) Rv=16.6WN−1.66

(4) $$n_{v\;}=I_{n}/4\pi \;R_{v}\;D\;\tau \;\left(P/4-I_{n}\right)$$

where *W_N_ = √FWN*^2^ − σ*^2^, *D* is the Ps atom diffusion coefficient, *τ* is the *p−Ps* lifetime, and *P* is theprobability of Ps formation in the polymer.

The parameters of the free volume–volume fraction of vacancies, of average vacancy sizewere determined by the positron annihilation (PA) method, in particular, by the method of angular correlation of paired annihilation gamma quanta [5].

Summarising the results shown in Table 2, it can be seen that all samples, irrespective of their degree of acetylation, are characterised by close *f* values between 0.011 and 0.016. This result should be regarded as natural, since all esters are in a glassy state when measured by the positron annihilation method. It is well known [7] that at the glass transition temperature (Table 1), all sorbents possess a single free volume value of 0.03. From the values of the free volume at 293 K compared to the free volume at the glass transition temperature, we estimate the range of the coefficient of thermal expansion of the free volume of cellulose ethers in the glassy state, which is on average α ≅ 1.4 × 10^−4^ cm^3^/g·K.

## 3. Results and Discussion

**Figure 2:**
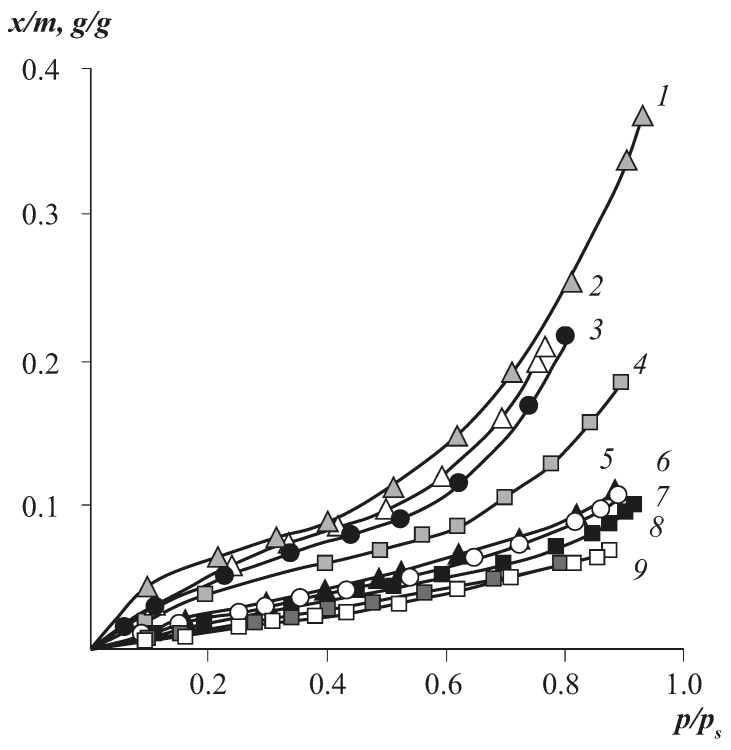
Isotherms of water vapour sorption by cellulose esters of different substitution degrees: 1—0, 2—0.6,3—1.21, 4—1.7, 5—2.00, 6—2.4, 7—2.7, 8—2.8, 9—3.00 at 30 °C.

**Figure 3:**
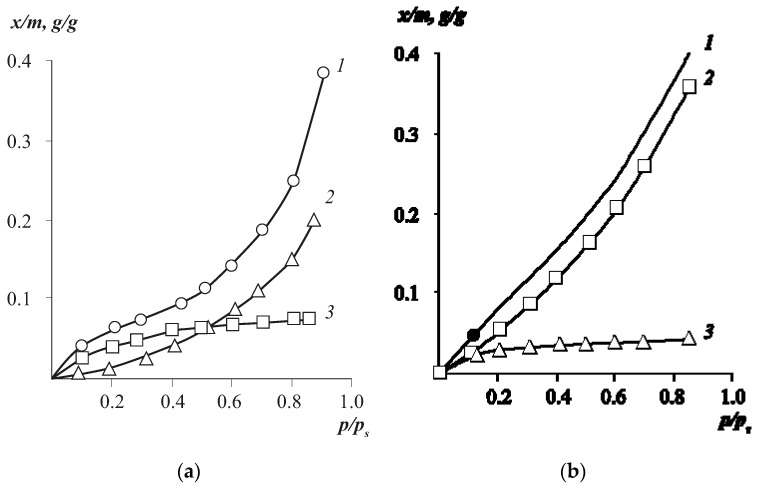
Decomposition of isotherms of water vapour sorption by cellulose hydrate at 20 °C (**a**) and cellulose acetate (SD = 2.00) at 20 °C (**b**) into components: 1—experimental isotherm, 2—Flory–Huggins isotherm, 3—Langmuir isotherm for (**a**,**b**).

Figure 2 and Figure 3 show typical isotherms of water vapour sorption by CA of different SD, which agree well with the literature data [22].

We can see that in contrast to unsubstituted cellulose hydrate (Figure 3), which has a pronounced S-shaped character, as the degree of substitution of hydroxyl groups with acetate fragments increases, there is a transition from S-shaped isotherms to concave-type isotherms. The sorption capacity of cellulose esters upon the transition of SD from 1 to 3 changes more than fourfold.

It is interesting to note that the transition from S-shaped to concave isotherms is also observed for cellulose hydrate, but during its thermal annealing, which is due to disequilibrium of the initial state of the sorbent and relaxation of excess free volume during annealing [9]. For cellulose esters, this effect is observed against the background of free volumeconstancy (Table 3). In contrast to cellulose hydrate, only weakly pronounced sorption–desorption hysteresis is observed for its esters, and sorption isotherms are characterised by high reproducibility, which we showed in repeated cycles of sorption–desorption. Thus, it can be stated that cellulose esters possess a stable supramolecular organisation with respect to water vapour sorption–desorption processes.

As it is known, the *S*-shaped sorption isotherm qualitatively indicates a complex mechanism of water dissolution in polymeric sorbents and, in particular, in cellulose esters. We consider the S-shaped isotherm within the framework of the double sorption model, where the S-shaped isotherm is considered as a result of superposition of two isotherms described by the Langmuir and Flory–Huggins equations [10,12]:

(5) $$\{\begin{array}{c} C=C_{1}+C_{2}\\ C_{1}=\frac{\varphi }{1-\varphi }\rho_{water/\rho_{polym.}}\\ \varphi =\ln\left(\frac{\varphi }{a}\right)+{\left(1-\varphi \right)}^{2}\chi +1\\ C_{2}=\frac{C_{H}^{\prime }Keq}{1+Keq}, \end{array}$$

where *C* is the total mass fraction of sorbed water, *C*_1_ is the mass fraction of dissolved water, *C*_2_ is the mass fraction of water sorbed according to the Langmuir mechanism, *ρ* is the sorbent density, K*_eq_* is the Langmuir equilibrium constant, and *φ* is the volume fraction of water sorbed by the polymer, *a* = *p*/*p_s_*.

The characteristic parameters of isotherms were calculated: the parameter of pair interaction *χ* and the limiting number of water molecules in the adsorption layer of the active center of the monomeric link of the macromolecule *C*_H_ (see Table 2). It should be noted that the Langmuir mode characterises the proportion of dissolved water molecules sorbed on the available active centers of cellulose ester macromolecules. As a rule, this process of sorbent molecule absorption is described by a convex isotherm.

The Flory–Huggins mode characterises dissolved molecules freely migrating in the volume. For them, the isotherm is concave relative to the abscissa axis. The results of decomposition of isotherms of water vapour sorption by cellulose hydrate (Figure 3a) and cellulose diacetate (Figure 3b) are presented as an example.

The sorption heats calculated from the temperature dependences for the Langmuir component [20] reach values of 14–16 kJ/mol; for the Flory–Huggins isotherms, they amount to 4–6 kJ/mol. This result once again confirms the assumption about the localised mechanism of water sorption on the CA polar active centers. Note that the obtained information contradicts the statement that “solid samples of cellulose and its esters completely lack free hydroxyl groups”.

**Figure 4:**
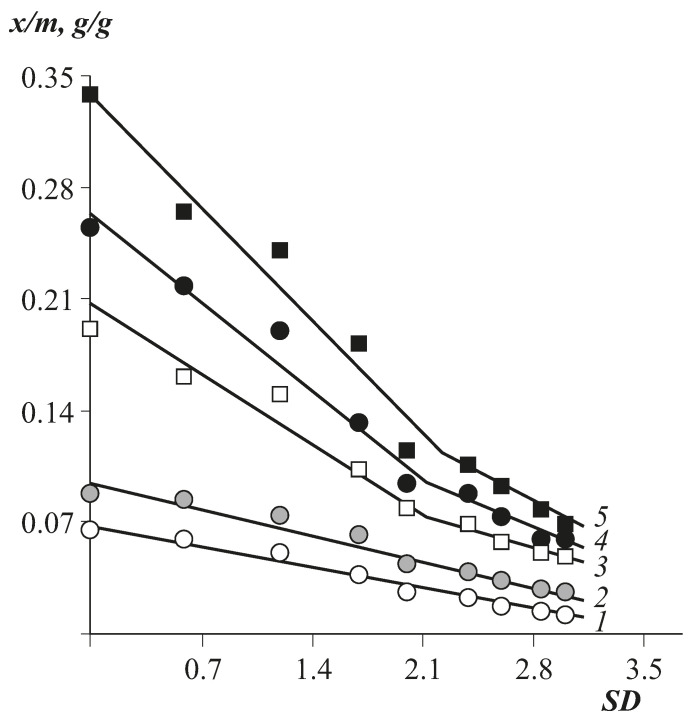
Dependence of sorption capacity of cellulose esters on the substitution degree at different *p*/*p_s_*: 1—0.2; 2—0.4; 3—0.7; 4—0.8; 5—0.9.

For CA with small and medium SD, the Langmuir component differs significantly in absolute value, while the Flory–Huggins component changes insignificantly. We attribute these differences in the behaviour of the Langmuir component to changes in the composition of the monomeric links of cellulose esters, namely, the content of hydroxyl groups. Indeed, despite the fact that the free volume parameters for all CAs are close, the fraction of hydroxyl groups with a high local sorption capacity is replaced by ester groups during esterification, the local sorption capacity for water vapour of which is several times lower than the sorption capacity of –OH groups. It is of fundamental importance that the availability of hydroxyl groups at all values of SD and ambient humidity remains unchanged, as evidenced by the linear dependence of the sorption capacity of esters on their SD (Figure 4 and Table 4).

Hydrate numbers of the ester group of the polymer monomeric link were calculated using sorption isotherms for mono-, di-, and triacetates of cellulose. The results of calculations in comparison with van Krevelen data are given in Table 5. We assume that the differences in the values of the ester group hydrate numbers are associated with the influence of the thermal prehistory of the sorbents and the ordering degree of the environment surrounding the sorption centers. Nevertheless, preliminary calculations showed that the obtained values of the hydrate numbers of the –COO– ester group in combination with the hydrate numbers of the van Krevelen hydroxyl group make it possible to calculate sorption isotherms for the monomeric units of cellulose esters of different composition.

**Figure 5:**
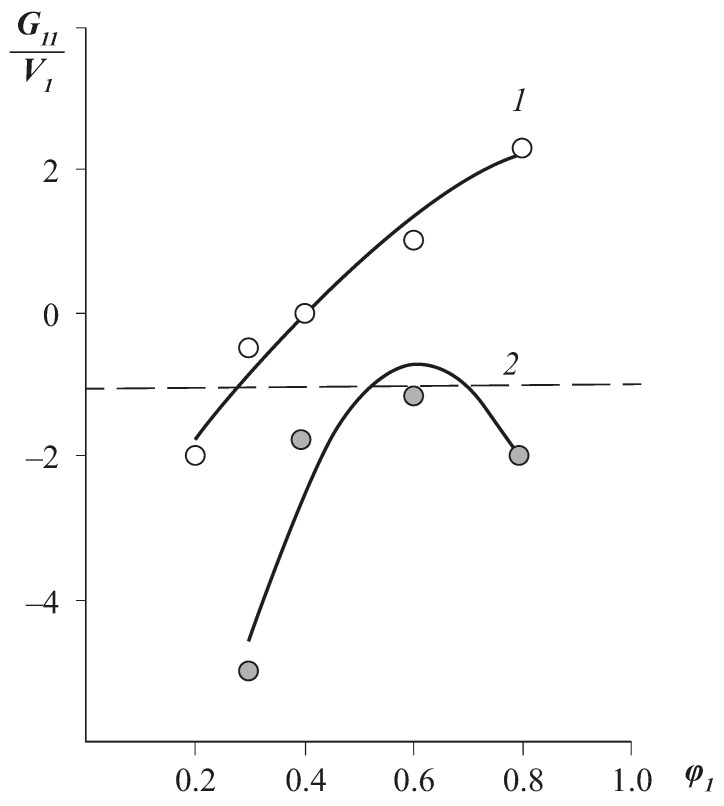
Integral of clustering of water molecules in the CA matrix with different degrees of substitution: 1—1.21; 2—2.6. The dotted line corresponds to *G*_11_/*v*_1_ = −1.

Another parameter characterising the state of the sorbed water molecules in the polymeric sorbent is related to the clustering integral *G*_11_*/v*_1_, which determines “the number of water molecules exceeding the average concentration of these molecules” [7]. The clustering integral was calculated using the equation:

(6) $$\frac{G_{11}}{v_{1}}=\;-\;\left(1-\varphi_{1}\right)\left[\frac{\partial \frac{a_{1}}{\varphi_{1}}}{\partial a_{1}}\right]-1$$

where *v*_1_ is the molar volume, *φ*_1_ is the volume fraction, and *a* is the water activity. As an example, Figure 5 shows the concentration dependences of the clustering integral for CA2 and CA6. It can be seen that for cellulose diacetate, the clustering exceeds the value *G*_11_/*v*_1_ of −1 already at vapour activity *p*/*p_s_* ≈ 0.4, while for triacetate *G*_11_/*v*_1_ ≤ −1, indicating the absence of clusters.

The total number of water molecules in the cluster *N_c_* calculated by the equation:

(7) $$N_{c}=-\varphi_{1}\left(\frac{G_{11}}{v_{1}}+1\right)+1$$

equals 3–4 in the area of high vapour activity.

This work is the first attempt to obtain experimental information on the changes in the free volume parameters of some of the studied polymers upon their filling by water molecules at different *p*/*p_s_*. We should note that already at the time of our studies, there was an original work [27], the authors of which used the same method of positron annihilation for qualitative identification of changes in the free volume of cured epoxy resins during their long exposure in water, that is, at only one fixed value of *p*/*p_s_* = 1. The main attention was paid to the correlation between the swelling kinetics and the kinetics of changes in the positron lifetime. We, on the other hand, in our work achieved the following:

- First, we performed measurements at different vapour activities, which allowed us Here are 21, pleaseto compare the data on the transformation of the free volume at different filling degrees with the sorption isotherms;
- Second, we obtained quantitative characteristics of free volume parameters of polymeric sorbents in isobar–isothermal equilibrium with the environment of different humidity.

**Figure 6:**
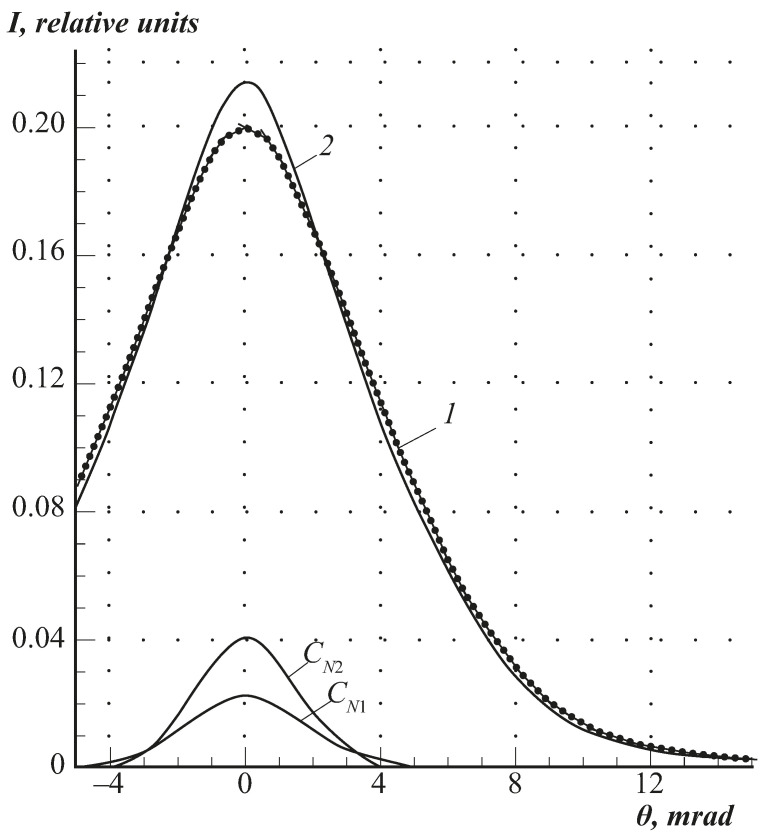
Angular correlation curves of annihilation scattering in cellulose acetate samples (degree of substitution 1.21): at *p*/*p_s_* 1—0.2, 2—0.7.

Figure 6 shows typical angular correlation (AC) curves for the annihilation measurements of CA samples. One can see that in the AC curves of cellulose esters, just as it is observed for most polymers [25,26], along with the broad component, there is a “narrow” component, the appearance of which is associated with the self-annihilation of the para Ps atoms localised in the micro cavities of free volume.

Table 2 presents the free volume parameters of the evacuated film samples of cellulose esters and the samples conditioned at different *p*/*p_s_*. It can be seen that the concentration of traps, the average radius of free volume microcavities R, and their volume fraction f for all CA samples are close. At the same time, as noted above, the sorption capacity of the samples differs quite significantly, especially with respect to the Langmuir component. Thus, for the samples with small substitution degree, there is a pronounced S-shape on the sorption isotherm, which we associated with the filling of the excess free volume by water molecules, while for CTA, the sorption isotherm has a purely Flory–Huggins character, and filling of the excess free volume according to the sorption measurements “does not occur”. This disagreement—the presence of excess free volume in the structure of the polymeric sorbent and the absence of Langmuir region on the sorption isotherm—can be reconciled by suggesting that the “surface” of the free volume microcavities contains different concentrations of the active centers—polar groups. For CA with a low substitution degree, the microcavity surface contains available free hydroxyl groups, whose hydration numbers (HN) reach values of 0.818 at *p*/*p_s_* = 0.9, whereas for CA with a high SD, such groups are virtually absent, and ester groups on the microcavity surface are characterised by comparatively low values of the HN of –COO–(0.14 at *p*/*p_s_* = 0.9).

**Figure 7:**
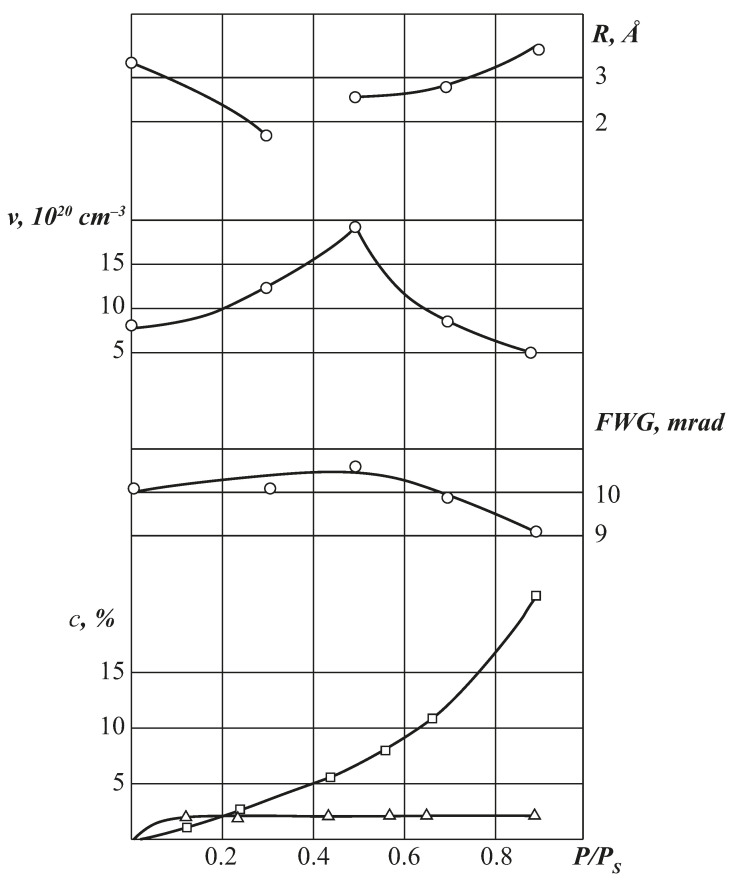
Changes in the parameters of free volume (*R, n_ν_*), matrix (FWG), and sorption characteristics of CA (SD = 1.21) at 20 °C as the sample is saturated with water. ∆—Langmuir component, □—Flory–Huggins isotherm.

When CA is saturated with water, the free volume parameters change continuously (Figure 7). In the region of low vapour activities (in the Langmuir region of the sorption isotherm), as the samples are saturated with water vapour, the average radius of microcavities R decreases due to the partial filling of the vacancy volume. An increase in the half-width of the wide CA component is observed, which indicates an increase in the total density of the polymer sorbent, and a decrease in the effective fraction of free volume. An unusual result for this region of sorbate activity is that the above changes in the parameters of the CA free volume are simultaneously accompanied by an increase in the concentration of traps per unit volume. This fact can be explained in the framework of the general concept of filling holes in the free volume with water molecules if we assume that microcavities have a fairly wide size distribution.

Thus, the simplest model of water sorption by cellulose ethers suggests that at low values of vapour activity and low degrees of acetylation, the double Langmuir + Flory-Huggins sorption model is realised. The Langmuir mode of sorbed water is most probably associated with the formation of hydrogen bonds on the accessible hydroxyl groups located on the “surface” of the vacancies of free volume. In the region of high activities, the formation of cluster structures is observed, which degenerate at the transition to di- and triacetates of cellulose. Along with the localised structures of sorbed molecules, there are free water molecules migrating through the polymer volume with partial self-diffusion coefficients 4.2 × 10^−9^ cm^2^/s in the AC2 matrix and 8.2 × 10^−9^ cm^2^/s in the TAC matrix. With increasing temperature, the diffusion coefficients increase. The calculated values of activation energy of water diffusion are 9–11 kcal/mol. The obtained data on hydrate numbers of hydroxyl groups and acetyl radicals make it possible to calculate a priori the sorption capacity of cellulose ethers in a wide range of relative humidities and to estimate the proportion of free hydroxyl groups acting as active centres at different sections of sorption isotherms.

This process terminates at the *p*/*p_s_* region, when the Langmuir sorption isotherm reaches its saturation. Then, when large holes are filled, the sorbate molecules are distributed near their surface, thereby reducing the effective radius of the microcavities. The result of this process is a change in the freevolume hole size distribution curve and, as a consequence, an increase in the trap concentration.

- In the region of high *p*/*p_s_*, where CA filling with sorbate molecules occurs predominantly by the Flory–Huggins mechanism, the character of changes in the freevolume parameters is different;
- Continuous increase of the mean radius of the freevolume holes with increasing *p/p_s_*;
- Decrease in the half-width of the broad AC component, indicating “loosening” of the polymer matrix at high values of humidity;
- Decrease in the concentration of positron traps.

Since this stage of the sorption process is reversible, in contrast to the initial Langmuir region, the study suggests that the observed changes in the free volume parameters are associated with an increase in the local mobility of macromolecular chain fragments. Since this process is observed at *T < T_g_* of the water-saturated samples, we assume that its appearance is related to the realisation of the *β*-mechanism of segmental mobility of the polymer.

## 4. Conclusions

It is interesting to note that for the majority of hydrophilic polymers under normal conditions of their storage and investigation by the positron annihilation methods, for which the average moisture content is 60%, the excess free volume of polymers is already filled with water, and only the second stage of their filling related to the plasticisation of the polymer is observed. This result should be taken into account both in the interpretation of positron annihilation data and in the theoretical description of the sorption process.

Thus, the simplest model of water sorption by cellulose esters suggests that at low values of vapour activity and low degrees of acetylisation, the double Langmuir + Flory-Huggins sorption model is realised. The Langmuir mode of sorbed water is most likely associated with the formation of hydrogen bonds on the available hydroxyl groups located on the “surface” of free volume vacancies. In the region of high activities, the formation of cluster structures is observed, which degenerate upon the transition to di- and triacetates of cellulose. Along with localised structures of sorbed molecules, there are free water molecules migrating through the polymer volume with partial coefficients of self-diffusion of 4.2 × 10^−9^ cm^2^/s in the CA2 matrix and 8.2 × 10^−9^ cm^2^/s in the CTA matrix. The diffusion coefficients increase with increasing temperature. The calculated activation energy of water diffusion is 9–11 kcal/mol.

## Figures and Tables

**Table 1:** Properties of the study objects.

Sample	Substitution Degree *γ*	Glass Transition Temperature, T_G_, K	Molar Mass, *M*_η_ × 10^−4^	Density, g/cm^3^	Solvent
CA1	0.6	503	7.7	1.29	DMF
CA2	1.21	493	7.5	1.27	DMF
CA3	1.70	473	6.9	1.30	DMF
CA4	2.03	457	7.8–8.8	1.32	DMF
CA5	2.40	-	8.2	1.33	DMF
CA6	2.60	462	7.5	1.30	DCM:ethanol 3:1
CA7	2.85–2.90	460	7.8–8.0	1.28	DCM:ethanol 9:1
CTA	3.0	456	6.8–7.2	1.27	DCM:ethanol 9:1

**Table 2:** Free volume parameters in CA films (T = 20 °C).

Sample	*p*/*p_s_*	Radius of the Spherical Vacancies, *R*, A°	Trap Concentration, *n_v_*, 10^20^ cm^−3^	Free Volume Fraction, *f* *
CA1 (SD = 0.6)	0	3.80	0.46	0.011
CA3 (SD = 1.70)	0	2.60	1.47	0.011
CA4 (SD = 2.00)	0	2.79	1.88	0.017
CA5 (SD = 2.40)	0	2.89	1.51	0.015
CA6 (SD = 2.60)	0	3.14	1.08	0.014
CA7 (SD = 2.90)	0	3.28	1.09	0.016
CA2 (SD = 1.21)	0	3.3	7.7	0.012
—”—	0.3	1.7	13.7	0.003
—”—	0.5	2.6	19.1	0.015
—”—	0.7	2.7	7.1	0.006
—”—	0.9	3.8	5.6	0.013

* The calculation is performed assuming that the positronium diffusion coefficient in the sample is 10^−4^ cm^2^/s.

**Table 3:** Free volume characteristics of water-free CA samples.

Designation	Free Volume Fraction, *f* *	Spherical Micropore Radius, R, A°
CA1	0.011	3.80
CA2	0.012	3.3
CA3	0.011	3.4
CA4	0.017	2.79
CA5	0.015	3.28
CA6	0.014	3.14
CA7	0.016	3.28
CTA	0.018	3.41

* The calculation is performed assuming that the positronium diffusion coefficient in the sample is 10^−4^ cm^2^/s.

**Table 4:** Characteristics of isotherms of water vapour sorption by CA.

Sorbent	Temperature, °C	*C*′_H_	*χ*
CA2, SD = 1.21	30	0.0248	1.06
	40	0.0112	1.12
CA3, SD = 1.71	20	0.0251	1.25
	30	0.0235	1.29
	40	0.0142	1.26
CA4, SD = 2.00	20	0.0083	1.34
	30	0.0083	1.49
	40	0.0131	1.43

**Table 5:** Molar water content in cellulose esters per polar group at different moisture contents at 25 °C.

Group	*p*/*p_s_* Values					
	0.2	0.5	0.7	0.8	0.9	1.0
–OH *	0.35	0.5	0.75	0.95	1.2	2.0
–C = O *	0.025	0.055	0.11	0.17	0.20	0.30
–COO– *	0.025	0.050	0.075	0.11	0.14	0.20
–COO– **	0.035	0.072	0.13	0.18	0.26	0.50

* van Krevelen data; ** our data.

## Data Availability

The study did not report any data.
